# The antimalarial screening landscape—looking beyond the asexual blood stage

**DOI:** 10.1016/j.cbpa.2019.01.029

**Published:** 2019-06

**Authors:** Sabrina Yahiya, Ainoa Rueda-Zubiaurre, Michael J Delves, Matthew J Fuchter, Jake Baum

**Affiliations:** 1 Department of Life Sciences, Imperial College London, Sir Alexander Fleming Building, Exhibition Road, South Kensington, London SW7 2AZ, UK; 2 Department of Chemistry, Imperial College London, Molecular Sciences Research Hub, White City Campus, Wood Lane, London W12 OBZ, UK; 3 London School of Hygiene and Tropical Medicine, Keppel Street, London WC1E 7HT, UK

## Abstract

In recent years, the research agenda to tackle global morbidity and mortality from malaria disease has shifted towards innovation, in the hope that efforts at the frontiers of scientific research may re-invigorate gains made towards eradication. Discovery of new antimalarial drugs with novel chemotypes or modes of action lie at the heart of these efforts. There is a particular interest in drug candidates that target stages of the malaria parasite lifecycle beyond the symptomatic asexual blood stages. This is especially important given the spectre of emerging drug resistance to all current frontline antimalarials. One approach gaining increased interest is the potential of designing novel drugs that target parasite passage from infected individual to feeding mosquito and back again. Action of such therapeutics is geared much more at the population level rather than just concerned with the infected individual. The search for novel drugs active against these stages has been helped by improvements to *in vitro* culture of transmission and pre-erythrocytic parasite lifecycle stages, robotic automation and high content imaging, methodologies that permit the high-throughput screening (HTS) of compound libraries for drug discovery. Here, we review recent advances in the antimalarial screening landscape, focussed on transmission blocking as a key aim for drug-treatment campaigns of the future.

**Current Opinion in Chemical Biology** 2019, **50**:1–9

This review comes from a themed issue on **Next Generation Therapeutics**

Edited by **Yimon Aye** and **Paul J Hergenrother**

For a complete overview see the Issue and the Editorial

Available online 12th March 2019

**https://doi.org/10.1016/j.cbpa.2019.01.029**

1367-5931/© 2019 The Authors. Published by Elsevier Ltd. This is an open access article under the CC BY license (http://creativecommons.org/licenses/by/4.0/).

## Introduction

Incredible progress has been made in reducing the global malaria burden since the declaration of the UN Millennium Development Goals in 2000. However, in recent years, progress has stalled, with incidence and death rates from malaria no longer declining [1]., Commitment to these goals triggered a spike in global funding and interest, resulting in an increased implementation of artemisinin combination therapies (ACTs), insecticide treated bed nets (ITNs) and indoor residual spraying (IRS) which was pivotal in addressing the global burden of malaria disease [2]. Parasite resistance to artemisinin, its derivatives and partner drugs [3] and mosquito resistance to insecticides are, therefore, key challenges to get reduction of malarial incidence back on track. It is increasingly acknowledged that a focus on innovation, and not just implementation of the current antimalarial armamentarium, is required to overcome these challenges [4^•^,^5^]. New drugs, with novel chemical structures and new modes of action (MoA), will likely be a key component of such innovation [5].

Many groups active in antimalarial drug discovery, coordinate their work within a framework of molecule type [target candidate profiles (TCP)], meaning the lifecycle stage which is compromised by the drug, and medicine class (target product profiles (TPP)], the final drug formulation defined as a combination of TCPs, developed by the not-for-profit Medicines for Malaria Venture, MMV [6^••^] (Table 1). Sought after characteristics include activity against asymptomatic stages (TCP3-5), endectocides targeting the mosquito (TCP-6) and symptomatic asexual blood stages (ABS), classified as TCP-1 (Table 1). Profiles meet different needs such as medicines for clinical case management, chemoprotection for travellers, and those aimed at breaking population transmission. Protection of the uninfected population is crucial for eventual local elimination of transmission, and can be achieved by either targeting the mosquito (via vector control, bite-prevention or endectocides) or via compounds with transmission blocking activity [6^••^]. One long-sought goal for optimal treatment formulation is the administration of a Single Encounter Radical Cure and Prophylaxis (SERCaP), removing blood parasitemia and the longer-lived parasite reservoir from patients for both radical cure and elimination of future transmission, all in one go [5].

**Table 1:** Classifications of Medicines for Malaria Venture TCP and TPPs

Target Candidate Profiles		
Profile	*Plasmodium* lifecycle stage target	Notes
TCP1	Asexual blood stages	Active against resistant strains of *Plasmodium*
Symptomatic treatment		
TCP3	Dormant liver-stage hypnozoites	Improved safety compared to primaquine and tafenoquine
Anti-relapse		
TCP4	Hepatic schizonts	Effective at equal/lower dose to TCP1 treatment
Chemoprotection		
TCP5	Gametocytes/Gametes	Low dose, less than TCP1 treatment
Transmission blocking		
TCP6	Insect vector (endectocides)	Low dose, less than TCP1 treatment
Transmission blocking		

Target Product Profiles		
Profile	TCPs Addressed	Notes
TPP1	TCP1	Single or multiple treatment medicines for treatment of: Severe malaria (TCP1)
	TCP3	Uncomplicated malaria and preventative treatment (TCP1)
Case Management	TCP5	Relapsing malaria (TCP3)
	TCP6	Asymptomatic stages for population protection (TCP5 & 6)

TPP2	TCP1	In the case of epidemics or for migratory populations
Chemoprotection	TCP4	

### Drug Discovery by screening

In recent years, great emphasis has been placed on high-throughput screening (HTS) of large compound libraries, to find novel therapeutics having a new MoA, combined with improvement of existing compounds through medicinal chemistry and structure activity relationship (SAR) studies. High-throughput screens (HTS) are generally categorised into two types: target-specific assays (usually biochemical) or whole-cell (phenotypic) tailored to meet the different TCP/TPP criteria [7]. Given the breadth in the literature of both, here we centre our discussion on phenotypic (specifically cellular) screens, with a particular focus on *P. falciparum*, the most virulent parasite causing malaria in humans [8].

Although *in vitro* culture of *P. falciparum* is routine, automation, liquid handling and high-throughput imaging have played key roles in recent advancements in HTS capacity [7]. This has been markedly helped by efforts from the chemical vendor industry and pharma to provide access of compound libraries to smaller institutions and academic research groups, permitting testing on a massive scale, often with millions of compounds. Combined with assay miniaturisation, this has led to development of robust, inexpensive, and reproducible screens, typically utilising 384- or 1536-well plate-based formats [7,9]. To date, the vast majority of screening campaigns have centred on ABS. Recently, however, this has expanded to transmission and pre-erythrocytic stages, including development of screening platforms for sporozoites [10], sexual stage gametocytes [11^••^], gametes [12], ookinetes [13] and liver stages [14]. In either context, parasite cultures are incubated with compounds of interest and parasite survival is determined as an assay readout. Structures and activity of antimalarial compounds derived from such phenotypic screens are then deposited in the chEMBL Neglected Tropical Disease archive [15].

### Asexual Blood Stage (ABS) screens

**Figure 1:**
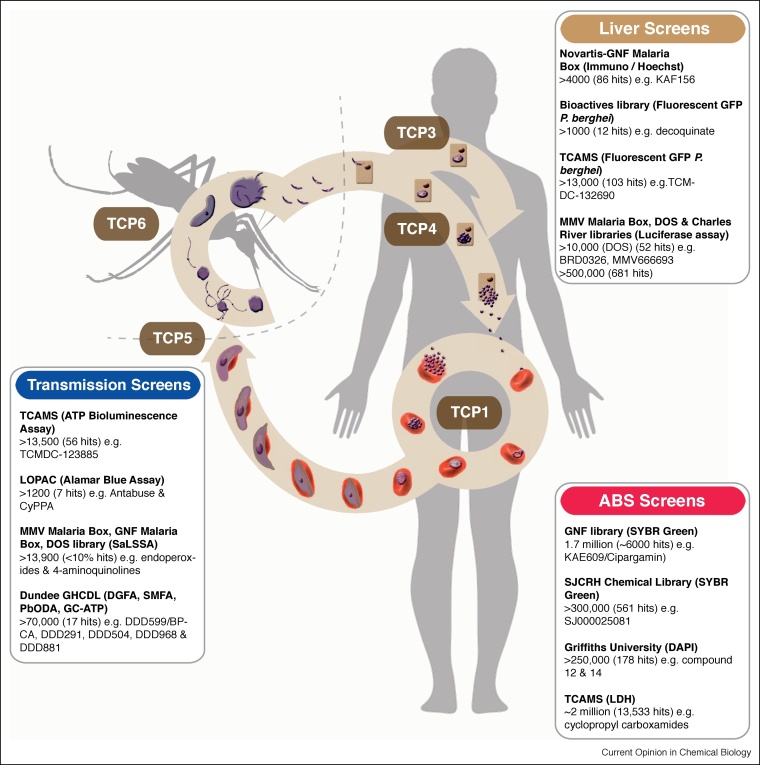
The Plasmodium parasite lifecycle highlighting notable cell-based screens and Target Candidate Profiles (TCP) for developmental drugs. The *Plasmodium* lifecycle occurs in stages between a mosquito vector and vertebrate host covering many different sites for drug intervention. Inoculation of motile sporozoites during the female *Anopheles* mosquito bloodmeal commences the asymptomatic liver stage. Exclusively to *P. vivax* and *P. ovale*, a proportion of liver-stage parasites form dormant hypnozoites (TCP3). Rupture of hepatic schizonts (TCP4) releases small merozoite forms that initiate the symptomatic stages (ABS, TCP1) made up of cycles of erythrocyte invasion, replication and release. A proportion of ABS parasites, rather than divide, commit to sexual differentiation to form the transmissible male and female gametocytes (TCP5), developing over 8–12 days (for *P. falciparum*), likely in the bone marrow, through morphologically distinct stages with sexual dimorphism most apparent at the mature stage V. Upon uptake to the mosquito during a bloodmeal, gametogenesis (formation of mature gametes), is induced rapidly (∼10–15 min). This follows environmental cues in the mosquito midgut, including a rise in pH, drop in temperature and the presence of xanthurenic acid, a mosquito-derived excretory product. Gametogenesis commences with the rounding up of both male and female gametocytes and their egress from the host erythrocyte. Male gamete formation, or exflagellation, is a remarkably rapid and tightly regulated process. The process includes three rounds of DNA replication alternating with endomitotic division, followed by the release of eight motile haploid male gametes. Fusion of male and female gametes ensues, leading to formation of a motile zygote that eventually colonizes the mosquito midgut, reseeding the vector for a new round of human infection [39]. Notable ABS cellular screens include those against the GNF Library; SJCRH (identifying hits with 50% inhibitory activity (IC50) of ≤2 μM); Griffiths University library (identifying hits for physicochemical and chemical diversity analysis) and TCAMS from GSK. Screens against the asymptomatic liver stages include screens of the Novartis-GNF Malaria Box (potent against ABS stages); bioactives library of commercially sourced compounds in clinical or pre-clinical development; TCAMS library (hits with dual blood and liver-stage activity) and the ultra-HTS of the MMV Malaria Box, DOS and most-recently Charles River libraries (hits with submicromolar exoerythrocytic stage activity). Transmission blocking screens to find drugs that block parasite transmission, compromising gametocyte or gamete viability, include those against the TCAMS library; LOPAC library using alamarBlue; MMV Malaria Box, GNF library and DOS library (using SaLSSA) and the Dundee GHCDL (using the DGFA).

**Figure 2:**
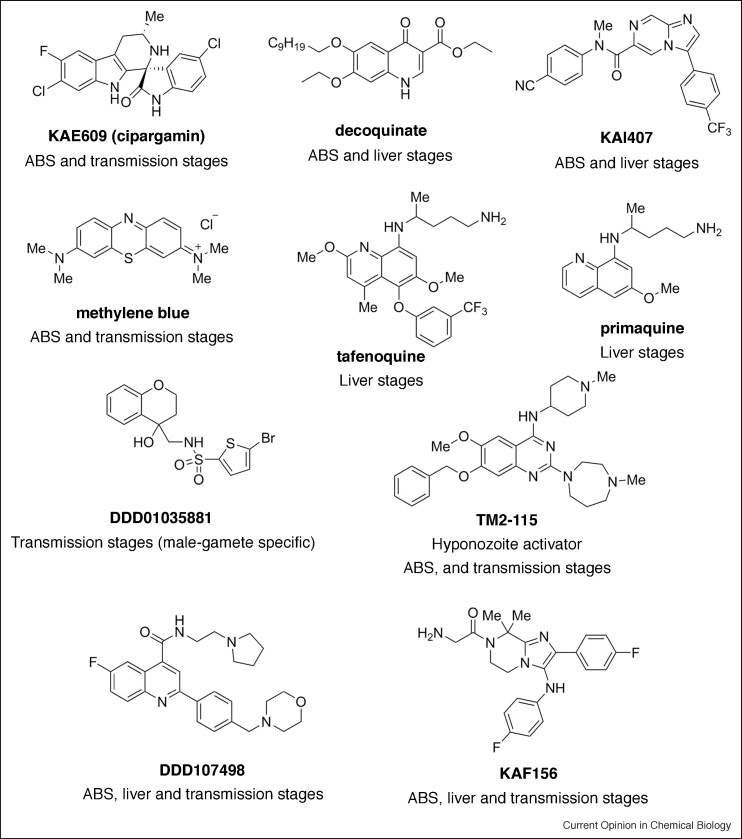
Notable frontline antimalarials with targets throughout the lifecycle. Selected antimalarials with activity against different stages of the parasite lifecycle. "Me" denotes methyl groups. KAE609 is a PfATP4 inhibitor which shows fast parasite clearance and transmission-blocking potential and is currently undergoing phase II clinical trials. Decoquinate is a dual-stage antimalarial (ABS and liver) with activity against the parasite mitochondrial bc1 complex. Primaquine and tafenoquine are the only liver-stage targeted compounds with the ability to kill hypnozoites in use despite their side effects. KAI407 is a hypnozoitocidal compound identified using *P. cynomolgi* sporozoites and primary monkey hepatocytes that targets the ABS and developing liver stage parasites as well. TM2-115 is a putative histone methyltransferase inhibitor with activity against both asexual and sexual stages, and the ability to induce dormant liver stages to resume their maturation. Methylene blue is a classical antimalarial showing transmission-blocking activity regardless of assay read-out and can thus be used as positive control in screening campaigns. DD01035881 is a male-gamete formation inhibitor identified from the GHCDL screen. KAF156 is a drug candidate with activity against ABS, liver and sexual stages, which was developed through the optimisation of a hit identified in one of the first liver stage screens. DDD107498 is a eEF2 inhibitor with activity across all parasite stages. Primaquine, Tafenoquine and DDD1035881 are used as racemates.

Novel compounds targeting asexual blood stages (under the umbrella of TCP-1) have long been seen as a priority in antimalarial research, being the causative agent of symptoms associated with malaria [6^••^]. The first *P. falciparum* ABS screen (indeed the first major HTS) tested 1.7 million compounds from the Genomics Institute of the Novartis Research Foundation (GNF) Chemical Library, identifying ∼6000 hits [16]. Similar screens followed using 300 000 compounds of the St. Jude Children’s Research Hospital (SJCRH) chemical library [17]; 250 000 compounds from the Griffiths University library [18]; and, possibly the largest study, from GlaxoSmithKline who screened almost 2 million compounds [19]. This latter screen yielded an enriched library of >13 500 future potential antimalarials, called the Tres Cantos antimalarial compound set (TCAMS) that has since seeded several other screens (for example, Refs. [20, 21, 22, 23]). The numerous asexual blood stage screens performed to date, each using very different methodologies (Figure 1), have identified thousands of hits, some of which have progressed to developmental antimalarials, such as the spirondolone KAE609 (Cipargamin, Figure 2) [24].

### Liver stage screens

Beyond ABS, in recent years, the search for novel antimalarials has pushed for drugs targeting other stages of the parasite lifecycle. *Plasmodium* hepatic forms have recently gained significant traction due to improved culture techniques, opening up possibilities for finding drugs with causal chemoprophylaxis against liver stages (TCP4) and those that may target the dormant hypnozoite stage (unique to *P. vivax* and *P. ovale* species) classified as TCP3 [25]. Targeting either form hits a natural bottleneck in the parasite lifecycle, and, therefore, a powerful way to reduce the probability of drug resistance developing [26]. One challenge remaining, however, is the need for complete parasite development in the mosquito, required to obtain infective sporozoites to seed assays, and the poor rates of *in vitro* hepatocyte infectivity. Relatively robust assays are starting to emerge for *in vitro* development [14,27], suggesting a turning point in liver stage screening studies. Liver stage screens are often focused on smaller libraries of commercially available compounds with known activity against the asexual blood stages.

One of the first liver-stage screens (Novartis-GNF Malaria Box) identified a lead imidazolopiperazine scaffold. This screen was performed using immunolabelled parasites to determine the ratio of parasitemia to host nuclei, using a high-content imaging system [28]. Lead optimisation yielded a drug candidate, KAF156 (Figure 2) [28], which is not only active against ABS and liver stages, but also blocks parasite transmission. KAF156 is currently undergoing clinical trials [29^••^]. Another focused screen tested 1037 existing drugs, also by high-content imaging, to detect fluorescent murine malaria parasite, *P. berghei,* liver stages, identifying decoquinate, a compound with activity against the parasite mitochondrial *bc_1_* complex [30]. Two additional screens worth noting used the TCAMS library, to identify 103 hits with dual inhibitory activity against blood and liver stages [23] and an ultra-HTS format luciferase-based assay, that tested both an open access library of small molecules with confirmed activity against *P. falciparum* ABS (the MMV malaria box) and a Diversity-Oriented Synthesis library from the Broad Institute [31] (Figure 1). Very recently, a landmark screen of half a million compounds from Charles River [66^••^], consisting of small molecules with an average weight of 369 Daltons, found more than 600 hits with sub-micromolar IC_50_s using a plate-based *P. berghei* assay (and validating assays with *P. vivax* and *P. falciparum*) similar to that developed in [31]. Hits included mitochondrial inhibitors and several others with potentially novel modes of action.

Although these screens have advanced the pre-erythrocytic targeting pipeline, at present, the only existing liver-stage targeted drugs in use and suited to targeting the hypnozoite stages are primaquine and tafenoquine (Figure 2). Both, however, are also associated with intravascular haemolysis in glucose-6-phosphate dehydrogenase (G6PD)-deficient patients [32]. The absence of an accepted *P. vivax* hypnozoite model is in part to blame for the limited anti-hypnozoite antimalarial discovery. The most robust platform for screening was, until recently, a low throughput *in vivo* imaging assay using *P. cynomolgi* and rhesus monkeys. An *in vitro* improvement to this using *P. cynomolgi* sporozoites and primary monkey hepatocytes [33] has successfully identified a hypnozoitocidal compound, KAI407 (Figure 2), that besides targeting the ABS is active against both liver developing parasites and hypnozoites [34]. Further advances in culture protocols and use of humanized mouse models add to the tool base towards the hoped-for radical cure treatment that would eliminate liver stages [35,36]. Although not yet adapted to an HTS format, one such assay [35,36] allowed for the identification of a compound, TM2-115, (Figure 2) a proposed *Plasmodium* histone methyltransferase inhibitor [37] with a unique “wake and kill” phenotype.

### Transmission blocking assays

Beyond liver stages, there is a growing awareness of the potential for targeting parasite transmission, diverting away from simply treating symptomatic (or pre-symptomatic) forms of parasite infection. Targeting transmission has long been seen as a critical step towards meeting the demanding goals of an eradication agenda [38]. Although billions of parasites may circulate an infected individual during asexual stages, only 0.2–1% are thought to commit to sexual development and, therefore, are responsible for transmission, constituting a massive lifecycle bottleneck [39]. This stage is permissive for transmission to the mosquito upon uptake of a blood meal, making them a viable transmission blocking drug target [39].

Drug targeting strategies focussed on transmission centre on two areas, either breaking transmission by targeting the mosquito vector itself (using endecticides such as Ivermectin [40^•^]), classified as TCP6 (recently reviewed in [41]), or targeting the *Plasmodium* sexual stages, blocking gametocyte or gamete development, classified as TCP5 [6^••^]. By stopping onward transmission, each is orientated towards protecting the wider population rather than the individual [42]. Although several platforms for discovering transmission blocking drugs have been developed (Figure 1), advances in screening for compounds targeting sexual development have been boosted by improvements to *in vitro* culture protocols for *P. falciparum* gametocytes [43^•^,44^•^,45^•^]. Being non-replicative developmental stages, however, gametocytes are not amenable to traditional DNA replication or cell proliferation markers, which has meant other measures of viability, including mitochondrial activity or fluorescent protein expression are required. Control compounds often used in such assays include classical antimalarials such as methylene blue (Figure 2), a compound in phase II trials which is consistently found to be active against transmission, though with ranging IC_50_ values (e.g. 12–490 nM). Artemisinin endoperoxides have generally proven inactive (>1 μM) against mature gametocytes.

One of the first gametocyte-centred screens used the MMV malaria box, aimed at the identification of dual asexual-sexually active drug candidates. Gametocytaemia was determined following expression of a transgenic gametocyte-specific protein pfs16-Luc-GFP marker, with cell viability determined using Mitotracker Red, a reporter of mitochondrial function [46]. Other studies have used similar strategies as a base for drug screening [47, 48, 49]. In parallel to these efforts, a group from GlaxoSmithKline developed a methodologically improved ATP bioluminescent assay, using reduction in ATP as a marker of cell injury and death [50]. Using this method, the group tested 17 gold-standard compounds with known antimalarial activity on purified stage IV—V gametocytes, before cytotoxicity and specificity tests with HepG2 cells. A follow-up screen examined dual activity of the TCAMS library against stage V gametocytes [51]. Towards an improved signal-to-noise ratio, required for HTS, several groups have developed colorimetric readouts for gametocyte viability. This includes parasite lactate dehydrogenase (pLDH) [52] and alamarBlue [53] as indicators of metabolic activity. Two recent large-scale screens are also worth highlighting, including the use of acridine orange (AO) to measure gametocytaemia and rounding-up post-activation as a marker of viability, adapted to 384-well format from researchers at the Istituto Superiore di Sanità in Rome [54] and, most recently, the Saponin-lysis Sexual Stage Assay (SaLSSA) from the University of California San Diego School of Medicine. [11^••^]. This latter assay utilises synchronised gametocytes and involves an *in situ* erythrocyte saponin-lysis before MitoTracker Red staining, highlighting parasites with an active mitochondrial membrane potential. Because it can work at low magnification, this automated high content imaging platform has been developed to 1534-well capacity and has been used effectively with several drug libraries [11^••^] (Figure 1).

### Sex specificity and viability

One of the key challenges, however, to drug discovery of the transmission stages, is the *in vivo* validation of hits. Most screens to date, validate any newly discovered hits with the Standard Membrane Feeding Assay (SMFA) [55] to determine onwards viability. Although widely considered the gold-standard for transmission blocking activity, the assay is extremely low-throughput. It involves treatment of gametocyte culture before feeding to malaria-susceptible *Anopheline* mosquitos using an artificial membrane. Mosquito midguts are then dissected 7–10 days after feeding and oocyst abundance is counted by microscopy to determine viability [56]. Attempts to increase throughput have been made and hold great promise if robustness and reproducibility in mosquito-feeds can be achieved [57]. What use of the SMFA demonstrates is that viability of the sexual stages is not the same thing as capacity to transmit (i.e. gametocytaemia does not equate with transmission). For example, it is clear that many exemplar transmission-blocking drugs like primaquine do not affect the presence of viable (but transmission-incompetent) gametocytes in peripheral blood [58^••^]. Meeting this challenge head on, a very different approach to transmission screening is to explore the effect of drugs not on gametocytes but on the developing gametes, mimicking their transformation in the mosquito midgut *ex vivo* without the limitations of the SMFA. *P. falciparum* notably has a female-biased sex ratio, with a range of ratios of between ∼3 and 5 females for every 1 male [59,60]. Combined with the fact that males exhibit an increased susceptibility to known antimalarials despite the greater abundance of females [59], has prompted development of assays that capture both male and female development independently. One of the most successful of these is the Dual Gamete Formation Assay (DGFA) [12,59], which measures male and female gametogenesis via automated imaging. Male gamete formation is signified by formation of ‘exflagellation centres’ as male gametes adhere to neighbouring erythrocytes; female gamete formation is detected by immunostaining of a surface protein expressed at the gamete surface upon egress. In measuring these two features, the assay provides a sex-specific gametocyte viability readout and has been developed to plate-based format [12]. Similar male-only [61] and female-only assays have also been developed [62]. Because of each entity’s focus on gamete formation, stage V gametocyte viability is encompassed in each assay since it is the only stage that will develop further upon triggering gametogenesis. The assay has proven to be a powerful high-throughput proxy for transmission and there is good evidence of a linear correlation between sex-specific gamete assays and SMFA activity [12,63]. However, a key caveat that remains with each of these assays is the viable production of *in vitro-*derived stage V *P. falciparum* gametocytes that are competent for exflagellation and onward transmission to mosquitoes [43^•^].

Advancement of the DGFA to 384-well plate format recently permitted an HTS of the University of Dundee Global Health Chemical Diversity Library (GHCDL), in which the DGFA was undertaken in parallel to ABS and other transmission blocking assays to discern compounds with varying activity profiles. The joint study between Imperial College London and GlaxoSmithKline [64^••^] is the largest transmission blocking focussed screen carried out to date on a non-biased library (i.e. a library unrelated to ABS activity). Numerous hits were identified displaying asexual-specific, dual asexual-sexual stage, sexual stage-specific and, for the first time, gamete-specific targeted activity. Of note, male specific, dual male-female gametocyte and male gamete only targeted compounds were also discovered. Among hits, several belonged to a cluster sharing an *N*-((4-hydroxychroman-4-yl)methyl)-sulphonamide scaffold, which shows promise for future transmission-only drug development (Figure 2).

The GHCDL screen, like many others, demonstrated the power of combining multiple platforms to find novel scaffolds with both new modes of action and multi-stage activity. A good example of the latter was the discovery of DDD107498, a translation elongation factor 2 (eEF2)-targeted compound (Figure 2) identified from the Dundee protein kinase scaffold library, which shows multiple activity against ABS, liver stages and male and female gamete formation [65]. Though many groups favour the clinical development of a multi-stage drug, one caution with this approach is the challenge that selection for parasite resistance will be amplified by its multiple points of sensitivity across the lifecycle (presuming it has a single pharmacological mode of action across the lifecycle). Combined formulations with drugs targeting different processes in different stages, in particular transmission, may be preferable in this case (as it has been with viral and bacterial infections), not only blocking transmission but protecting partner drugs from resistance development [64^••^].

## Conclusions

Application of HTS technologies to the liver and the sexual stages of *Plasmodium* are receiving an increasing interest as a necessary addition to efforts in antimalarial drug discovery. Innovation in technologies and novel modes of action becomes increasingly important in an era of emerging ACT resistance and the plateau in declining malaria incidence. Advances in screening for drugs that act along each step of the parasite lifecycle (from ABS to transmission and back again) have advanced significantly in recent years with development of assays testing activity at each stage. Though these phenotypic screens exhibit clear advantages over target-based approaches in their scope, they also raise challenges in drug mode of action identification. However, by combining forces with medicinal chemistry to undertake detailed SAR of hits, the prospect of developing new lifecycle orientated drugs with new modes of action becomes increasingly feasible.

## References and recommended reading

Papers of particular interest, published within the period of review, have been highlighted as:

• of special interest
•• of outstanding interest

## References

[1] (2017). World malaria report 2017. Press Release.

[2] Bhatt S., Weiss D.J., Cameron E., Bisanzio D., Mappin B., Dalrymple U., Battle K.E., Moyes C.L., Henry A., Eckhoff P.A. (2015). The effect of malaria control on plasmodium falciparum in Africa between 2000 and 2015. Nature.

[3] Anthony M.P., Burrows J.N., Duparc S., Moehrle J.J., Wells T.N. (2012). The global pipeline of new medicines for the control and elimination of malaria. Malar J.

[4•] Griffin J.T., Bhatt S., Sinka M.E., Gething P.W., Lynch M., Patouillard E., Shutes E., Newman R.D., Alonso P., Cibulskis R.E., Ghani A.C. (2016). Potential for reduction of burden and local elimination of malaria by reducing plasmodium falciparum malaria transmission: a mathematical modelling study. Lancet Infect Dis.

[5] Rabinovich R.N., Drakeley C., Djimde A.A., Hall B.F., Hay S.I., Hemingway J., Kaslow D.C., Noor A., Okumu F., Steketee R. (2017). Malera: an updated research agenda for malaria elimination and eradication. PLoS Med.

[6••] Burrows J.N., Duparc S., Gutteridge W.E., Hooft van Huijsduijnen R., Kaszubska W., Macintyre F., Mazzuri S., Mohrle J.J., Wells T.N.C. (2017). New developments in anti-malarial target candidate and product profiles. Malar J.

[7] Flannery E.L., Chatterjee A.K., Winzeler E.A. (2013). Antimalarial drug discovery - approaches and progress towards new medicines. Nat Rev Microbiol.

[8] White N.J., Pukrittayakamee S., Hien T.T., Faiz M.A., Mokuolu O.A., Dondorp A.M. (2014). Malaria. Lancet.

[9] Hovlid M.L., Winzeler E.A. (2016). Phenotypic screens in antimalarial drug discovery. Trends Parasitol.

[10] Hegge S., Kudryashev M., Smith A., Frischknecht F. (2009). Automated classification of plasmodium sporozoite movement patterns reveals a shift towards productive motility during salivary gland infection. Biotechnol J.

[11••] Plouffe D.M., Wree M., Du A.Y., Meister S., Li F., Patra K., Lubar A., Okitsu S.L., Flannery E.L., Kato N. (2016). High-throughput assay and discovery of small molecules that interrupt malaria transmission. Cell Host Microbe.

[12] Ruecker A., Mathias D.K., Straschil U., Churcher T.S., Dinglasan R.R., Leroy D., Sinden R.E., Delves M.J. (2014). A male and female gametocyte functional viability assay to identify biologically relevant malaria transmission-blocking drugs. Antimicrob Agents Chemother.

[13] Delves M.J., Ramakrishnan C., Blagborough A.M., Leroy D., Wells T.N., Sinden R.E. (2012). A high-throughput assay for the identification of malarial transmission-blocking drugs and vaccines. Int J Parasitol.

[14] Roth A., Maher S.P., Conway A.J., Ubalee R., Chaumeau V., Andolina C., Kaba S.A., Vantaux A., Bakowski M.A., Luque R.T. (2018). A comprehensive model for assessment of liver stage therapies targeting plasmodium vivax and plasmodium falciparum. Nat Commun.

[15] Gaulton A., Hersey A., Nowotka M., Bento A.P., Chambers J., Mendez D., Mutowo P., Atkinson F., Bellis L.J., Cibrian-Uhalte E. (2017). The chembl database in 2017. Nucleic Acids Res.

[16] Plouffe D., Brinker A., McNamara C., Henson K., Kato N., Kuhen K., Nagle A., Adrian F., Matzen J.T., Anderson P. (2008). In silico activity profiling reveals the mechanism of action of antimalarials discovered in a high-throughput screen. Proc Natl Acad Sci U S A.

[17] Guiguemde W.A., Shelat A.A., Bouck D., Duffy S., Crowther G.J., Davis P.H., Smithson D.C., Connelly M., Clark J., Zhu F. (2010). Chemical genetics of plasmodium falciparum. Nature.

[18] Avery V.M., Bashyam S., Burrows J.N., Duffy S., Papadatos G., Puthukkuti S., Sambandan Y., Singh S., Spangenberg T., Waterson D., Willis P. (2014). Screening and hit evaluation of a chemical library against blood-stage plasmodium falciparum. Malar J.

[19] Gamo F.J., Sanz L.M., Vidal J., de Cozar C., Alvarez E., Lavandera J.L., Vanderwall D.E., Green D.V., Kumar V., Hasan S. (2010). Thousands of chemical starting points for antimalarial lead identification. Nature.

[20] Crowther G.J., Hillesland H.K., Keyloun K.R., Reid M.C., Lafuente-Monasterio M.J., Ghidelli-Disse S., Leonard S.E., He P., Jones J.C. (2016). Biochemical screening of five protein kinases from plasmodium falciparum against 14,000 cell-active compounds. PLoS One.

[21] Miguel-Blanco C., Molina I., Bardera A.I., Diaz B., de Las Heras L., Lozano S., Gonzalez C., Rodrigues J., Delves M.J., Ruecker A. (2017). Hundreds of dual-stage antimalarial molecules discovered by a functional gametocyte screen. Nat Commun.

[22] Gomez-Lorenzo M.G., Rodriguez-Alejandre A., Moliner-Cubel S., Martinez-Hoyos M., Bahamontes-Rosa N., Gonzalez Del Rio R., Rodenas C., Fuente J., Lavandera J.L., Garcia-Bustos J.F., Mendoza-Losana A. (2018). Functional screening of selective mitochondrial inhibitors of plasmodium. Int J Parasitol Drugs Drug Resist.

[23] Raphemot R., Lafuente-Monasterio M.J., Gamo-Benito F.J., Clardy J., Derbyshire E.R. (2015). Discovery of dual-stage malaria inhibitors with new targets. Antimicrob Agents Chemother.

[24] White N.J., Pukrittayakamee S., Phyo A.P., Rueangweerayut R., Nosten F., Jittamala P., Jeeyapant A., Jain J.P., Lefevre G., Li R. (2014). Spiroindolone kae609 for falciparum and vivax malaria. N Engl J Med.

[25] Campo B., Vandal O., Wesche D.L., Burrows J.N. (2015). Killing the hypnozoite-drug discovery approaches to prevent relapse in plasmodium vivax. Pathog Glob Health.

[26] Mazier D., Renia L., Snounou G. (2009). A pre-emptive strike against malaria’s stealthy hepatic forms. Nat Rev Drug Discov.

[27] March S., Ng S., Velmurugan S., Galstian A., Shan J., Logan D.J., Carpenter A.E., Thomas D., Sim B.K., Mota M.M. (2013). A microscale human liver platform that supports the hepatic stages of plasmodium falciparum and vivax. Cell Host Microbe.

[28] Meister S., Plouffe D.M., Kuhen K.L., Bonamy G.M., Wu T., Barnes S.W., Bopp S.E., Borboa R., Bright A.T., Che J. (2011). Imaging of plasmodium liver stages to drive next-generation antimalarial drug discovery. Science.

[29••] White N.J., Duong T.T., Uthaisin C., Nosten F., Phyo A.P., Hanboonkunupakarn B., Pukrittayakamee S., Jittamala P., Chuthasmit K., Cheung M.S. (2016). Antimalarial activity of kaf156 in falciparum and vivax malaria. N Engl J Med.

[30] da Cruz F.P., Martin C., Buchholz K., Lafuente-Monasterio M.J., Rodrigues T., Sonnichsen B., Moreira R., Gamo F.J., Marti M., Mota M.M. (2012). Drug screen targeted at plasmodium liver stages identifies a potent multistage antimalarial drug. J Infect Dis.

[31] Swann J., Corey V., Scherer C.A., Kato N., Comer E., Maetani M., Antonova-Koch Y., Reimer C., Gagaring K., Ibanez M. (2016). High-throughput luciferase-based assay for the discovery of therapeutics that prevent malaria. ACS Infect Dis.

[32] Watson J., Taylor W.R.J., Bancone G., Chu C.S., Jittamala P., White N.J. (2018). Implications of current therapeutic restrictions for primaquine and tafenoquine in the radical cure of vivax malaria. PLoS Negl Trop Dis.

[33] Dembele L., Gego A., Zeeman A.M., Franetich J.F., Silvie O., Rametti A., Le Grand R., Dereuddre-Bosquet N., Sauerwein R., van Gemert G.J. (2011). Towards an in vitro model of plasmodium hypnozoites suitable for drug discovery. PLoS One.

[34] Zeeman A.M., van Amsterdam S.M., McNamara C.W., Voorberg-van der Wel A., Klooster E.J., van den Berg A., Remarque E.J., Plouffe D.M., van Gemert G.J., Luty A. (2014). Kai407, a potent non-8-aminoquinoline compound that kills plasmodium cynomolgi early dormant liver stage parasites in vitro. Antimicrob Agents Chemother.

[35] Dembele L., Franetich J.F., Lorthiois A., Gego A., Zeeman A.M., Kocken C.H., Le Grand R., Dereuddre-Bosquet N., van Gemert G.J., Sauerwein R. (2014). Persistence and activation of malaria hypnozoites in long-term primary hepatocyte cultures. Nat Med.

[36] Mikolajczak S.A., Vaughan A.M., Kangwanrangsan N., Roobsoong W., Fishbaugher M., Yimamnuaychok N., Rezakhani N., Lakshmanan V., Singh N., Kaushansky A. (2015). Plasmodium vivax liver stage development and hypnozoite persistence in human liver-chimeric mice. Cell Host Microbe.

[37] Malmquist N.A., Moss T.A., Mecheri S., Scherf A., Fuchter M.J. (2012). Small-molecule histone methyltransferase inhibitors display rapid antimalarial activity against all blood stage forms in plasmodium falciparum. Proc Natl Acad Sci U S A.

[38] Sinden R.E. (2017). Developing transmission-blocking strategies for malaria control. PLoS Pathog.

[39] Angrisano F., Tan Y.H., Sturm A., McFadden G.I., Baum J. (2012). Malaria parasite colonisation of the mosquito midgut--placing the plasmodium ookinete centre stage. Int J Parasitol.

[40•] Smit M.R., Ochomo E.O., Aljayyoussi G., Kwambai T.K., Abong’o B.O., Chen T., Bousema T., Slater H.C., Waterhouse D., Bayoh N.M. (2018). Safety and mosquitocidal efficacy of high-dose ivermectin when co-administered with dihydroartemisinin-piperaquine in kenyan adults with uncomplicated malaria (ivermal): a randomised, double-blind, placebo-controlled trial. Lancet Infect Dis.

[41] Chaccour C.J., Kobylinski K.C., Bassat Q., Bousema T., Drakeley C., Alonso P., Foy B.D. (2013). Ivermectin to reduce malaria transmission: a research agenda for a promising new tool for elimination. Malar J.

[42] Leroy D., Campo B., Ding X.C., Burrows J.N., Cherbuin S. (2014). Defining the biology component of the drug discovery strategy for malaria eradication. Trends Parasitol.

[43•] Delves M.J., Straschil U., Ruecker A., Miguel-Blanco C., Marques S., Dufour A.C., Baum J., Sinden R.E. (2016). Routine in vitro culture of p. Falciparum gametocytes to evaluate novel transmission-blocking interventions. Nat Protoc.

[44•] Brancucci N.M., Goldowitz I., Buchholz K., Werling K., Marti M. (2015). An assay to probe plasmodium falciparum growth, transmission stage formation and early gametocyte development. Nat Protoc.

[45•] Duffy S., Loganathan S., Holleran J.P., Avery V.M. (2016). Large-scale production of plasmodium falciparum gametocytes for malaria drug discovery. Nat Protoc.

[46] Duffy S., Avery V.M. (2013). Identification of inhibitors of plasmodium falciparum gametocyte development. Malar J.

[47] Adjalley S.H., Johnston G.L., Li T., Eastman R.T., Ekland E.H., Eappen A.G., Richman A., Sim B.K., Lee M.C., Hoffman S.L., Fidock D.A. (2011). Quantitative assessment of plasmodium falciparum sexual development reveals potent transmission-blocking activity by methylene blue. Proc Natl Acad Sci U S A.

[48] Buchholz K., Burke T.A., Williamson K.C., Wiegand R.C., Wirth D.F., Marti M. (2011). A high-throughput screen targeting malaria transmission stages opens new avenues for drug development. J Infect Dis.

[49] Peatey C.L., Spicer T.P., Hodder P.S., Trenholme K.R., Gardiner D.L. (2011). A high-throughput assay for the identification of drugs against late-stage plasmodium falciparum gametocytes. Mol Biochem Parasitol.

[50] Lelievre J., Almela M.J., Lozano S., Miguel C., Franco V., Leroy D., Herreros E. (2012). Activity of clinically relevant antimalarial drugs on plasmodium falciparum mature gametocytes in an atp bioluminescence “transmission blocking” assay. PLoS One.

[51] Almela M.J., Lozano S., Lelievre J., Colmenarejo G., Coteron J.M., Rodrigues J., Gonzalez C., Herreros E. (2015). A new set of chemical starting points with plasmodium falciparum transmission-blocking potential for antimalarial drug discovery. PLoS One.

[52] D’Alessandro S., Silvestrini F., Dechering K., Corbett Y., Parapini S., Timmerman M., Galastri L., Basilico N., Sauerwein R., Alano P., Taramelli D. (2013). A plasmodium falciparum screening assay for anti-gametocyte drugs based on parasite lactate dehydrogenase detection. J Antimicrob Chemother.

[53] Tanaka T.Q., Williamson K.C. (2011). A malaria gametocytocidal assay using oxidoreduction indicator, alamarblue. Mol Biochem Parasitol.

[54] Lucantoni L., Silvestrini F., Signore M., Siciliano G., Eldering M., Dechering K.J., Avery V.M., Alano P. (2015). A simple and predictive phenotypic high content imaging assay for plasmodium falciparum mature gametocytes to identify malaria transmission blocking compounds. Sci Rep.

[55] Churcher T.S., Blagborough A.M., Delves M., Ramakrishnan C., Kapulu M.C., Williams A.R., Biswas S., Da D.F., Cohuet A., Sinden R.E. (2012). Measuring the blockade of malaria transmission--an analysis of the standard membrane feeding assay. Int J Parasitol.

[56] Li T., Eappen A.G., Richman A.M., Billingsley P.F., Abebe Y., Li M., Padilla D., Rodriguez-Barraquer I., Sim B.K., Hoffman S.L. (2015). Robust, reproducible, industrialized, standard membrane feeding assay for assessing the transmission blocking activity of vaccines and drugs against plasmodium falciparum. Malar J.

[57] Vos M.W., Stone W.J., Koolen K.M., van Gemert G.J., van Schaijk B., Leroy D., Sauerwein R.W., Bousema T., Dechering K.J. (2015). A semi-automated luminescence based standard membrane feeding assay identifies novel small molecules that inhibit transmission of malaria parasites by mosquitoes. Sci Rep.

[58••] Dicko A., Brown J.M., Diawara H., Baber I., Mahamar A., Soumare H.M., Sanogo K., Koita F., Keita S., Traore S.F. (2016). Primaquine to reduce transmission of plasmodium falciparum malaria in mali: A single-blind, dose-ranging, adaptive randomised phase 2 trial. Lancet Infect Dis.

[59] Delves M.J., Ruecker A., Straschil U., Lelievre J., Marques S., Lopez-Barragan M.J., Herreros E., Sinden R.E. (2013). Male and female plasmodium falciparum mature gametocytes show different responses to antimalarial drugs. Antimicrob Agents Chemother.

[60] Robert V., Sokhna C.S., Rogier C., Ariey F., Trape J.F. (2003). Sex ratio of plasmodium falciparum gametocytes in inhabitants of dielmo, senegal. Parasitology.

[61] Lozano S., Gamallo P., Gonzalez-Cortes C., Presa Matilla J.L., Fairhurst R.M., Herreros E., Amaratunga C., Rodrigues J. (2018). Gametocytes from k13-propeller mutant plasmodium falciparum clinical isolates demonstrate reduced susceptibility to dihydroartemisinin in the male gamete exflagellation inhibition assay. Antimicrob Agents Chemother.

[62] Miguel-Blanco C., Lelievre J., Delves M.J., Bardera A.I., Presa J.L., Lopez-Barragan M.J., Ruecker A., Marques S., Sinden R.E., Herreros E. (2015). Imaging-based high-throughput screening assay to identify new molecules with transmission-blocking potential against plasmodium falciparum female gamete formation. Antimicrob Agents Chemother.

[63] Colmenarejo G., Lozano S., Gonzalez-Cortes C., Calvo D., Sanchez-Garcia J., Matilla J.P., Leroy D., Rodrigues J. (2018). Predicting transmission blocking potential of anti-malarial compounds in the mosquito feeding assay using plasmodium falciparum male gamete inhibition assay. Sci Rep.

[64••] Delves M.J., Miguel-Blanco C., Matthews H., Molina I., Ruecker A., Yahiya S., Straschil U., Abraham M., Leon M.L., Fischer O.J. (2018). A high throughput screen for next-generation leads targeting malaria parasite transmission. Nat Commun.

[65] Baragana B., Hallyburton I., Lee M.C., Norcross N.R., Grimaldi R., Otto T.D., Proto W.R., Blagborough A.M., Meister S., Wirjanata G. (2015). A novel multiple-stage antimalarial agent that inhibits protein synthesis. Nature.

[66••] Antonova-Koch Y., Meister S., Abraham M., Luth M.R., Ottilie S., Lukens A.K., Sakata-Kato T., Vanaerschot M., Owen E., Rodriguez J.C.J. (2018). Open-source discovery of chemical leads for next-generation chemoprotective antimalarials. Science.

